# Injured Nerve Regeneration using Cell-Based Therapies: Current Challenges

**Published:** 2015

**Authors:** E. S. Petrova

**Affiliations:** Federal State Budgetary Scientific Institution «Institute of Experimental Medicine», St. Petersburg, Akad. Pavlov str.,12, 197376, Russia

**Keywords:** nerve regeneration, nerve, cell therapy, stem cells

## Abstract

This paper reviews the recent research progress in the past several years on promoting peripheral nerve recovery using stem and progenitory cells. The emphasis is placed on studies aimed at assessing various stem cells capable of expressing neurotrophic and growth factors and surviving after implantation in the nerve or a conduit. Approaches to improving nerve conduit design are summarized. The contribution of stem cells to axonal regeneration and neural repair is discussed. The side effects associated with cell-based treatment are highlighted. From the studies reviewed, it is concluded that the fate of transplanted stem cells needs further elucidation in a microenvironment-dependent manner.

## INTRODUCTION

Re-growth of peripheral nerve fibers could be induced using different approaches. Historically, enhanced nerve regeneration has been promoted by the administration of drugs [1], physical factors (magnetic field) [2, 3], and electrical stimulation [4-6]. Nerve surgery [7] and the microsurgical suture technique [8] have witnessed impressive development in recent years. Another option is bioartificial structures (conduits) that could serve as an alternative to autologous nerve grafts aimed at bridging the defects in nerve continuity. However, these approaches have failed to produce an efficient tool for nerve repair. A possible explanation is that, despite extensive studies by A.Waller, S.Ramon y Cajal, and Doynikov B.S. in the field of nerve regeneration [9-11], the molecular mechanisms underlying posttraumatic processes in nerve fibers remain poorly understood and require further investigation.

Following crushing or transaction injuries, degeneration takes place at a distance from the site of the injury, including axon degeneration, myelin breakdown and removal, and macrophage infiltration. All these events are collectively known as Wallerian degeneration. Within hours of nerve injury, axonal regeneration occurs: re-growth of nerve fibers proceeds from the proximal nerve segment. Scar tissue forms at the site of the lesion, obstructing axonal guidance, which results in traumatic neuroma formation. In addition, the degenerative processes lead to poor re-innervation of the target tissue or organ. These challenges emphasize the need for the development of new therapeutic strategies for stimulating nerve regeneration.

An important role in axonal re-growth is played by the humoral factors that provide a microenvironment for the guidance of axonal sprouts. This list includes growth and neurotrophic molecules, cytokines, and extracellular matrix proteins [12-14]. To study their effect on nerve repair, several models have been proposed: targeted delivery of growth factors into the injured nerve or conduit using microcapsules or osmotic minipumps (infusion pumps for continuous administration of test agents); application of nerve grafts or plasmids expressing neurotrophic and angiogenic factors [15-18].

A promising approach to fostering nerve regeneration is cell therapy, whereby trophic and growth factors are provided by engrafted cells such as syngeneic Schwann cells (neurolemmocytes) [12, 13, 15]. Following a traumatic injury, it is the Schwann cells that form the myelin sheath and produce such factors as NGF,VEGF,BDNF and other molecules that promote nerve repair. However, the production of viable donor Schwann cells in a desired concentration sometimes fails.

In the past decade, cell therapy for nerve defects has progressed to the use of embryonic stem cells, MSC, olfactory cells, stem cells of hair follicles, and other stem cells alongside donor Schwann cells. The findings of these studies are reviewed in [19-25]. However, the growing body of studies carried out in Russia and abroad raises new questions that have to be addressed. The objective of this study was to review papers on nerve repair published over the last three years.

It is important to highlight the current challenges in this field: (i) selection of cells capable of production of neurotrophic and growth factors and long-term survival when engrafted at the lesion site or conduit, (ii) investigation of the mechanisms behind the growth and regeneration of nerve fibers, (iii) improvement of conduits and their luminal fillers, (iv)development of efficient therapeutic strategies using stem cells, and (v) evaluation of nerve regeneration following injury and cell therapy. Few studies ask questions with regard to the potential side effects brought about by treatment.

The effect of cell therapy on nerve reconstruction could be evaluated using different models. Cells are administered into the injured nerve or conduit bridging the proximal and distal stumps intravenously or intramuscularly [26]. Nerve defect models have been developed and successfully used: nerve crush with forceps [27-31], nerve ligation injury [32], and nerve transection followed by approximation [33]. Of particular interest are studies involving synthetic conduits for the repair of nerve gaps. Over the past several years, bioabsorbable materials for fabricating conduits and luminal fillers have become the focus of much research. Such materials will offer optimal microenvironments for graft survival [20, 34-36].

## CELL TYPES USED IN CELL THERAPY FOR NERVE REGENERATION

Current research on nerve regeneration is generally performed on mesenchymal stem cells (MSC) derived from bone marrow [37], adipose tissue [24, 25, 38-42], umbilical cord stroma [43], and amniotic fluid [44]. The choice of bone marrow or fat tissue is guided by the fact that they provide an easy access and autologous source to stem cells for transplantation therapies. In addition, MSC have the capacity to modulate immune responses. Recently, MSC were shown to display immunosuppressive activity [40, 45]. The analysis of the molecular mechanisms underlying the interaction between MSC and immune cells demonstrated that MSC suppress T and B lymphocytes and inhibit dendric cell maturation [46]. However, this is not in agreement with the findings of McGrath *et al*. [37], who found that, in a rat sciatic nerve injury model, MSC combined with a fibrin glue conduit promote axon regeneration only when exposed to immunosupressive treatment with cyclosporine A. Three weeks postoperatively, macrophage and lymphocyte infiltration was decreased following cyclosporine A administration, which promoted axonal re-growth. Conflicting findings regarding the effects of MSC on immune responses can be explained by the origin of stem cells and the route of delivery. *In vitro *studies demonstrated that MSC derived from bone marrow, fat tissue, and umbilical cord have various effects on antibody production by B-cells [47]. It is also shown that MSC from different sources differ in plasticity, neuronal differentiation potential, and paracrine activity [48, 49].

Another study has noted the migratory capacity of MSC [50]. MSC enhance axon regeneration not only when delivered to the injured nerve or conduit bridging the nerve gap [26, 39, 51], but also when administered intravenously [27, 30, 52]. The MSC migration potential makes possible their detection at the site of sciatic nerve injury on day 7 post intravenous injection to mice and enhance functional recovery of the sciatic nerve [27].

Along with MSC, neuronal stem cells (NSC) are also used in cell therapy. Lin *et al. *[53] harvested spinal cord-derived NSC from 14- to 15-day rat embryos and cultured them for 7 days in a differentiation medium. When differentiated into cells with neuronal and glial phenotypes (the onset of βIII-tubulin or GFAP production, respectively), they were implanted into sectioned distal tibial nerves. The engraftment restored function in the denervated rat gastrocnemius muscle. Similar experiments were performed in the past [54]; however, the novelty of the work by Lin *et al*. [53] lies in the time point at 7 d post transection they discovered optimal for cell transplantation. After several days post nerve injury, the acute phase of inflammation is over, proinflammatory cytokines are fewer, and Schwann cells start to proliferate and produce trophic factors [53]. This milieu is attractive to implanted NSC rather than the nerve milieu immediately after a trauma. ;

In several studies, axon regeneration in mice was evaluated using NSC derived from the subventricular zone of adult mice [55]. This approach makes possible the survival of motor neurons in the spinal cord of the host undergoing retrograde degeneration after a sciatic nerve injury. In addition, it results in a 3-fold increase in the number of regenerating myelinated axon fibers distal to the site of nerve defect. It is suggested that NSC, as well as MSC, have immunomodulatory properties [55].

Researchers from the USA [56] and Japan [57] working independently carried out experiments with induced pluripotent stem cells (iPSCs) for neural tissue engineering. For a brief time, iPSCs derived from somatic cells of an adult organism (in particular, skin fibroblasts) through gene activation were widely used for cell therapy studies. This is because of the high proliferative potential of these cells and their easy accessibility. Bioabsorbable conduits seeded with these cells improve axon regeneration several fold versus controls [57]. This effect is much more pronounced when using bioabsorbale conduits seeded with iPSCs and bFGFloaded microspheres [58]. The use of NSC derived from human iPSCs showed that engrafted cells contribute in the distal segment of the injured nerve among nerve fibers [56]. The human nuclear antigen (NuMA) was utilized to detect the engrafted cells in histological examinations [56]. Concurrent identification of NuMA and the Schwann cell specific marker S100β allowed researchers to conclude that transplanted cells differentiate into neurolemmocytes. More importantly, the mechanism by which axon growth is accelerated is linked to the contribution of the engrafted NSC cells to myelin sheath formation. There is evidence that in various nerve injury models iPSCs have the capacity to differentiate into different cell types: for example, neurons positive for βIII-tubuline [59] or vascular smooth muscle cells [60]. Finally, allowing for the findings of these pioneering studies, it is concluded that the fate of iPSCs in the engrafted microenvironment is complicated and requires further investigation.

Raheja *et al. *[33] attempted bone-marrow-derived mononuclear cells (BM-MNC) in a rat sciatic nerve injury model. A month post inoculation, the outcome of nerve regeneration was found to be cell dose-dependent. Unfortunately, the authors did not speculate on the possible reasons for this observation. Likely, this is the result of BM-MNC paracrine activity reported previously [61]. It cannot be ruled out that transplanted MNC are engaged in rapid clearance of myelin debris, thus enhancing regenerative outgrowth.

The search for more effective regenerative options led to the discovery of a new cell type derived from skeletal muscle – muscle-derived stem/progenitor cells [31, 62, 63]. Tamaki *et al*. [63] demonstrated that after implantation into the nerve stump these precursor cells are capable of differentiating into Schwann cells, endoneurial and perineurial cells, as well as blood vessel cells (endotheliocyte, perycites, smoth muscle cells). Their availability and easy accessibility favor their use for autologous grafting, but there are findings reporting side effects associated with the use of these cells [62].

## STUDYING THE MECHANISMS BEHIND THE EFFECT OF REGENERATIVE THERAPIES ON PERIPHERAL NERVE REPAIR

The factors influencing the successful outcome of cell therapy on nerve repair are poorly understood and could be classified into the following: (i) differentiation toward the Schwann cell lineage, (ii) contribution to myelination of regenerating axons, (iii) production of trophic factors and extracellular matrix proteins that provide a milieu for axonal outgrowth, (iv) stimulation of proliferation and differentiation of endogenous cells, (v) stimulation of angiogenesis, and (vi) immunosupression in the injured nerve.

**Differentiation of stem cells toward the Schwann cell phenotype**

It remains debatable whether Schwann-like cells derived from implanted stem cells can be successfully used. There is a hypothesis positing that transplanted stem cells can directly differentiate into Schwann cells and facilitate axonal myelination. By contrast, an alternative hypothesis states that this cannot take place without prior *in vitro *transdifferentiation or predifferentiation of stem cells. The term predifferentiation refers to the use of NSC or ENC. The term transdifferentiation applies to the use of MSC. Although a more appropriate word would be transdetermination, proposed by V.E Okhotin *et al. *[64]. It is known that* in situ *MSC have the potential to differentiate along mesodermal lineages: bone, muscle, adipose tissue, etc; they are determined to differentiate into their particular cell types. Culturing of MSC in chemically defined media induces a switch in lineage commitment, known as transdetermination, toward neurolemmocytes, neurons, astrocytes, and other cell types.

Tomita *et al*. [28] performed *in vitro *and *in vivo *studies of glial differentiation of MSC derived from human adipose tissue. It was established that following exposure to glial growth factors MSC transdifferentiate into a Schwann cell phenotype. Lineage-committed MSC demonstrated a 7-fold higher survival rate after implantation than multipotent MSC in a rat tibial nerve injury study. In addition, transdifferentiated MSC (labeled with GFP) contribute to axon myelination: approximately 30% of engrafted cells were integrated in the myelin sheath of the regenerating axons. They were positive for GFP and P0, the marker of neurolemmocytes. The ability of MSC to differentiate toward a Schwann cell phenotype has been observed by others [65, 66].

Another hypothesis suggests that transdetermination of MSC into Schwann cells does not take place, and that rather they remain in their uncommitted state [67]. In contrast, therapy-associated axon growth is enforced by MSC production of trophic factors rather than their transdifferentiation [26, 67, 68].

**Stem cell production of trophic factors and extracellular matrix molecules**

In the past three years, many studies pertaining to nerve repair have been conducted with stem cells of different origins: bone marrow, adipose tissue, umbilical cord blood, etc. Stem cells have gained research interest as a promising source of biochemical mediators [69]. The capacity for producing a broad spectrum of trophic factors, growth factors, and cytokines has been well established [27, 38, 70, 71]; however, in an age-dependent manner in humans and animals [72, 73].

The most potent trophic factors secreted by MSC and used in regenerative therapies are NGF, BDNF, NF-3, and insulin-like growth factor-1 (IGF-I) [24, 27, 29, 38, 51]. Furthermore, MSC produce angiogenic factors such as VEGF and platelet-derived growth factor (PDGF) [27, 38].

There is convincing evidence that adipose-derived stem cells release the brain-derived neurotrophic factor (BDNF) and promote axonal regeneration. It was found that antibody-based neutralization of BDNF has an inhibitory effect on axonal recovery [29]. Using an antibody assay, it was demonstrated that MSC introduced into the conduit, which bridges the nerve defect, express bFGF [71].

Unfortunately, the mechanisms and cellular events governed by stem-cell-secreted factors are not fully elucidated. Fairbairn *et al. *suggest that these neurotrophic molecules target sensory and motor neurons [26]. Transplanted stem cells at the injury site mediate a retrograde neuroprotective effect on adjacent motor and sensory neurons, thereby increasing axon numbers. MSC delivered to the injured nerve prevent neuronal loss in the dorsal root ganglia by producing BDNF, endothelial growth factor, hepatocyte growth factor, and the insulin-like growth factor [70].

A positive outcome in stem-cell-based therapy is observed 1 week following surgery [29]. It is know that peripheral nerve injury induces axonal degeneration and demyelination in the distal stump [9, 74, 75]: nerve transection leads to complete degeneration of the nerve fibers of the distal segment of the injured nerve, whereas crush injury allows to preserve some axons. In this regard, it is tempting to speculate that the presence of implanted stem cells with paracrine activity at the site of the injury in the very early days promotes axonal survival rather than re-growth.

**Stimulation of endogenous host cells**

Endogenous Schwann cells create a growth-permissive environment for nerve reconstruction. These are cells capable of producing trophic factors, cytokines and extracellular matrix proteins required for axonal maintenance and regeneration [12-14]. Cell therapy is thought to activate endogenous Schwann cells. Incorporation of stem cells into the injured nerve or a bridging conduit upregulates the proliferation of local Schwann cells and secretion of bioactive molecules [51, 71, 76, 77]. The administration of NSC enhances NGFandHGF expression in these Schwann cells [77]. Marconi *et al. *[27] investigated the effect of a conditioned medium on cultured MSC and found that under *in vitro *conditions MSC produce a range of neuroprotective factors, except for the glial-derived neurotrophic factor(GDNF). Interestingly, GDNF levels were detected in the injured nerve of mice treated with MSC. A possible explanation could be that endogenous Schwann cells are stimulated by engrafted MSC to express GDNF in the local milieu.

Improved axonal recovery in response to cell therapy is associated with elevated angiogenesis. A recent study reported that the outcome of axonal regeneration depends on the vascular supply and perfusion [78]. Genetic studies showed that angiogenic factors enhance nerve reconstruction [16, 17]. Improved blood supply to the nerve with a lesion is due to the fibroblast growth factor, endothelial growth factor, placenta growth factor, and other angiogenic molecules released by MSC [38]. Adipose-derived stem cells seeded into a fibrin nerve conduit improve capillary formation in the tube and facilitate nerve regeneration by expressing VEGF-A and angiopoietin-1 [79].

**Inhibition of connective tissue and scar formation**

The inflammatory responses and fibrosis processes induced thereof impede axonal invasion. It is considered that regenerative strategies ameliorate these consequences. Marconi *et al. *[27] used a rat model of sciatic nerve crush injury to assess axonal degeneration in the distal segment after administration of MSC. The expression of the T-lymphocyte marker CD3 and the monocyte/macrophage marker CD11b+was down-regulated at the injured nerve on the 7, 14, and 21 days post administration of MSC. The engrafted stem cells modulate the immune response and modify the microenvironment [46]. Hsu *et al*. [80] used a chitosan- laminin scaffold filled in a silicone conduit and tested in a rat sciatic nerve injury model. In addition, this conduit was seeded with endogenous bone marrow- derived MSC. A histological analysis indicated that the area around the tube wall was characterized by prominent eosinophil and macrophage infiltration, whereas treatment with MSC reduced the extent of the inflammation within the injured region. The axon growth-enhancing effect of MSC seems to be due to anti- inflammatory cytokines. The range of cytokines-produced MSC is reviewed in [81].

There is evidence that neural stem-cell-based therapy is an option for mitigating the inflammatory response in an injured nerve after surgery. It was shown that the levels of inflammatory cytokines (interleukin 1 and 6) are decreased following NSC treatment [52]. Unfortunately, understanding of the role of cytokines in axonal degeneration and recovery remains frustratingly poor: therefore, the use of stem cells requires fundamental research.

## TISSUE-ENGINEERED CONDUITS FOR PERIPHERAL NERVE REGENERATION

In clinical practice, axonal regeneration across >3 cm gaps is achieved by autologous nerve grafting. Treatment- associated adverse effects (neuromas) and limited accessibility of donor grafts prompted a search for conduits to replace autologous tissues (reviewed in [20, 35, 80, 82, 83]). The classification of conduits to bridge nerve defects is well presented in the recent review paper [35].

Current research is focused on resorbable constructs and luminal fillers for axonal guidance [84]. Candidate constructs should meet the following criteria: biocompatibile, porous, and biodegradable with nontoxic degradation products.

Experimental conduits can be filled with collagen, fibrin, laminin, hydrogel, and keratin. There are strategies to promote a favorable microenvironment in the conduit for axonal growth by localized release of growth factors (fibroblast growth factor, VEGF, neurotrophins (NF-3, NGF), neuropoietic cytokines) and stem cell delivery. Growth factors guide axonal growth into implanted tubes and promote Schwann cell proliferation, which creates a permissive microenvironment for nerve reconstitution.

Another important criterion for conduits is to support the differentiation and survival of supportive cells seeded within the lumen [59, 85].

Although conduits can be biocompatible, nontoxic and resorbable, however, they fail to promote stem cell survival. For example, polycaprolactone conduits are hydrophobic and prevent cell adhesion, whereas poly-(lactic-co-glycolic acid) conduits release inhibitory byproducts for cell proliferation upon degradation [80]. Besides being a permissive conduit for survival of bone-marrow-derived MSC, the chitosan-laminin scaffold has drawbacks. It was found that chitosan breakdown products cause chronic inflammatory damage [80].

Alongside the development of synthetic tubes to bridge nerve gaps, a search is currently underway for novel nerve guidance conduits. Blood vessels have been widely used as biologic tubes [52, 86]. Importantly, even in the past [74], successful outcomes were achieved with blood vessels as artificial nerve guides. A recent study evaluated the potential of using xenographic conduits to promote axonal re-growth [87]. As nerve guidance conduits, acellular nerve, artery, and dermis were assessed in a rodent model. The nerve, artery, and dermal tissue were decellularized, leaving extracellular matrix proteins. Histological evaluation of the extracellular matrix content showed that all decellularized conduits were positive for collagen types I, III, and IV, fibronectin and laminin in various combinations. The artery conduit rich in collagen types I and IV and laminin but negative for collagen type III and fibronectin outperformed the other two types of conduits and permitted axonal re-growth and myelination.

There are works with the use of acellular nerve conduits and stem cells. These conduits carry basal membranes and collagen fibers and enhance cell proliferation, migration, and adhesion. Furthermore, they facilitate the survival of transplanted stem cells [22, 67, 88, 89]. To ensure cell viability within an acellular conduit, it is important to consider the procedure used for decellularization. A variety of decellularization approaches for nerve gap repair are summarized in [22], where cold temperature preservation, chemical detergent decellularization, and irradiation are discussed.

It has been demonstrated that acellular nerve xenografts combined with bone marrow stromal cells cause neither rejection nor inflammation and have axonal growth-promoting properties in a rat model [88]. After implantation, conduit-seeded cells differentiate into Schwann-like cells. Expression of NGF, BDNF, and other factors by these cells creates a microenvironment similar to endoneurium. Electrophysiological evaluation of nerve conductivity and morphological examination of regenerated nerves demonstrated that nerve regeneration and functional recovery was equal between xenogeneic and autologous acellular nerve graft transplants (the autologous nerve grafting technique is the gold standard for peripheral nerve reconstruction) [88].

In summary, a wide spectrum of conduits has become available. The outcome of engrafted stem cells is determined by conduit materials and scaffolds. Moreover, the survival and differentiation of cells seeded within a nerve conduit require further study. There is an opinion that the majority of transplanted cells fail to survive, which has prompted efforts to elevate cell viability.

## STRATEGIES TO IMPROVE THE EFFICACY OF REGENERATION THERAPY FOR NERVE DAMAGE

In the past several years, new approaches have been introduced to enhance cell therapies for nerve reconstitution. For example, concomitant transplantation of MSC and adjacent cells has been reported [90]. Co-cultivation of MSC and lemmocytes directs the differentiation of MSC towards a Schwann cell lineage, with lemmocytes producing much higher levels of neurotrophic factors as compared with neurolemmocytes. The use of MSC in combination with Schwann cells for conduit seeding proved to be more efficient for axonal regeneration than conduits seeded with either single cell suspension [90].

Laser irradiation has been a useful tool in axon repair using a biodegradable conduit [91, 92]. The positive effect is likely due to inhibition of inflammatory responses in the injured nerve.

Using various models, the combination of biochemical mediators seeded on a nerve conduit has proved instrumental in promoting transplanted cell viability, thereby stimulating axonal growth. Luo *et al. *reported the use of MSC in combination with TGF-β1 to ameliorate the apoptotic cell death of transplanted cells [93]. As a consequence, enhanced angiogenesis and decreased immune response contribute to successful nerve repair [93]. A poly-lactic-co-glycolic-acid nerve conduit loaded with NSC and neurotrophin-3 (NF-3) improves engrafted cell survival. NF-3 provides a permissive microenviroment for NSC to survive and differentiate mainly towards neurons [35]. Another study demonstrated the potential for combining stem cells with substance P for enhancing nerve recovery in the skin [94].

The combined use of bone-marrow-derived stromal cells and chondroitinase ABC (a bacterial enzyme used to treat herniated discs) was found superior to stem cell monotherapy in terms of augmenting nerve regeneration and preventing implanted cell death [95]. A possible mechanism is that chondroitinase ABC digests the chondroitin sulfate which is involved in connective tissue scar formation at the injured site.

## EVALUATION OF THE DEGREE OF NERVE REGENERATION AFTER TREATMENT

All experimental studies are concerned with the appropriate evaluation of the extent of axonal outgrowth. In our opinion, morphometric parameters such as semithin transverse sections of nerves or conduits have emerged as a reliable diagnostic indicator of nerve regeneration. This method has been widely applied in regenerative studies [4, 32, 36, 51, 91, 94].

Immunostaining can also be carried out to quantify nerve fibers using antibodies to axonal markers such as βIII-tubulin [37, 79] or neurofilaments (NF) [36, 51, 57], the P0 protein [75] or the Schwann cell marker myelin [28]. A modern method based on calculating the area covered by structures containing Schwann cell or axonal markers has been reported [51, 57].

Immunohistochemical techniques allow one to evaluate the degree of nerve regeneration by transverse sections of the nerve. Longitudinal sections of the nerve can also be used for quantification. The length of regenerating axons is calculated using the neuronal growth cone protein GAP-43 [27], PGP 9.5, a broad neural marker expressed in nerve fibers and the neurons of the peripheral nervous system [68] and the axonal marker βIII-tubulin [79].

Alongside a morphometric evaluation, physiological tests have been used for the assessment of regenerative success [28, 30, 42, 54, 67] and nerve conductivity [39, 53, 60, 75, 96].

Another approach to assessing nerve recovery is to look at the retrograde degeneration of the motor neurons and sensory neurons of spinal ganglia following the nerve injury [40, 52, 79, 97]. Using a sciatic nerve injury rat model, a laminin-coated chitosan conduit seeded with MSC was shown to suppress cell death of motor neurons in the lumbar spinal cord and improved axonal outgrowth several fold with regard to the non-seeded conduit [80]. It is also true that cell seeded polycaprolactone scaffolds attenuate retrograde degeneration of the neurons of spinal ganglia in rats with a sciatic nerve injury [70]. Of note, the neuroprotective effect is associated with conduits primed with stem cells pre-differentiated towards a Schwann cell phenotype [70]. There is evidence to suggest that embryonic neural tissue allografted into the injured site supports the survival of sensory neurons [98].

Nerve regeneration could be evaluated by measuring the weight and the area of the innervated muscle and its structural characteristics. Complete functional nerve regeneration is impeded by structural changes in the target tissues after denervation. For example, sciatic nerve transaction leads to gastrocnemius atrophy. The extent of nerve repair was evaluated by weighing the innervated muscle, a histological analysis of muscle fibers, and immunohistochemical staining of pre-and postsynaptic terminals at nerve muscle synapses [34, 54, 65, 86, 96, 99].

In 2012, a few papers were published concerning the use of *in vivo *MRI monitoring for assessing nerve regeneration after injury and MSC transplantation [100, 101]. An advantage of MRI is the capacity for tracking the fate of transplanted cells labeled with a superparamagnetic iron oxide nanoparticle [41, 102].

## ADVERSE EFFECTS ASSOCIATED WITH CELL THERAPY

The possible adverse effects of cell therapy have been widely discussed [103-108]. Negative consequences include host immune response to non-self stem cells, tumor development, inflammation and connective tissue scar formation, disturbance of gut microflora, etc. This issue of side effects following embryonic and adult stem cell delivery to the injured nerve has also been raised in [62, 85, 109].

A careful review of the literature published in the past several years suggests a paucity of studies addressing the issue of stem-cell-related tumorogenesis, because embryonic stem cells were discarded for their highest tumorogenic capacity compared to all other stem cells. NSC can only be used after pre-differentiation, for example, into neural and glial cells, which reduces tumor initiation.

The search for suitable stem cells could yield unexpected findings. Lavasani *et al. *[62] reported the use of murine muscle-derived stem/progenitor cells (MDSPC) for repairing nerve defects. It was shown that these cells are able to generate neurospheres and undergomyogenic, neuronal, and glial differentiation, expressing lineage-specific markers. After differentiation, MDSPC generate large neoplastic growths 11 weeks post-implantation.

Simultaneously, MDSPC were implanted into gastrocnemius muscles, where they underwent normal myogenic differentiation into myocytes. This finding highlights the importance of microenvironment-specific transformation.

Successful outcomes seem to be restricted to the use of pre-differentiated stem cells, whereas uncommitted MSC can lead to detrimental consequences [85, 110]. The risk of tumor development should be assessed in long-term studies. Unfortunately, in the field of regenerative therapy such observations are scarcely found. Some follow-up studies have reported no side effects at 12 months post transplantation [56, 111].

Importantly, much attention has been focused on clarifying the relationship between MSC and derived tumor cells, because there are plenty of pathways implicated in stem-cell-dependent tumor progression. There is evidence suggesting a role for the angiogenic, growth promoting, and immunosupressive effects of MSC in maintaining neoplastic growth [112, 113]. However, MSC are capable of tumor suppression [69, 114]. Long-term co-culturing of murine embryonic MSC derived from bone marrow with U251MGglioma cells changes the effect of MSC on tumor cells in a time-dependent fashion: at the early stage of culturing MSC promote tumor cell division, followed by tumor cell division suppression [115]. The relationship between MSC and tumor cells is being investigated; however, current knowledge is limited. Further work is needed to reach an unambiguous conclusion.

Along with papers providing experiential evidence of the negative outcomes of cell therapy, there is a study that reports an insignificant or even unobservable effect after cell therapy [116]. It is likely that the effect is short-term as previously described for stem cells evaluated in other experimental models [108,117]. These issues need to be explored in future.

## CONCLUSION

Numerous studies pertaining to the development of new regenerative therapies for nerve reconstitution show the effectiveness of stem cell treatment for axonal outgrowth and conductivity recovery. Unfortunately, the mechanism by which endogenous and exogenous stem cells contribute to regenerative success or failure remains poorly understood. Further studies are also required to identify the factors mediating the interaction between implanted stem cells and host cells, such as Schwann cell, macrophages, vessel cells, loose connective tissue cells, and epi- and perineurial cells.

The most recent studies were based on MSC derived from different sources: bone marrow, adipose tissue, cord blood, etc. The intrinsic property of these cells to produce biochemical mediators could promote the regenerative process after a traumatic injury. In addition, these cells permit autologous transplantation.

The findings obtained with experimental animals in the last decade have been extended to clinical trials [118-121]. Importantly, in 2012, positive effects of cell-based therapy were first reported using an animal model of diabetic polyneuropathy [60,93].

Despite numerous published studies, the fate of transplanted stem cells and precursor cells remains an issue of limited knowledge [39, 85, 122]. This is particularly important given the application of the novel materials used as conduits to bridge a gap between proximal and distal nerve stumps. The conduit provides a permissive microenvironment for cell survival and differentiation of transplanted cells. Conduits alone fail to promote transplanted cell survival and engraftment without additional therapeutic approaches.

The search is still on for means of providing directional guidance to regenerating axons. The cell-based approaches recently reported raise a wide array of questions that need to be addressed. To ensure safe medical techniques, it is important to accumulate data on the pre-differentiation and trans-determination of engrafted cells, which would clarify the mechanisms whereby engrafted stem cells facilitate regeneration. To reduce the side effects associated with cell therapies, the fate of implanted stem cells and precursor cells should be clearly defined for a period comparable with the lifespan of laboratory animals. The design of conduits and luminal fillers should be refined in terms of the microenvironment they provide for survival, differentiation, and functioning of implanted cells.

## Abbreviations

PNS: peripheral nervous system
SC: stem cells
NGF: nerve growth factor
VEGF: vascular endothelial growth factor
BDNF: brain-derived neurotrophic factor
bFGF: basic fibroblast growth factor
HGF: hepatocyte growth factor
NF-3: neurotrophin-3
MRI: Magnetic Resonance Imaging
GFP: green fluorescent protein
MSC: mesenchymal stem cells
